# Healthcare migration in Italian paediatric haematology-oncology centres belonging to AIEOP

**DOI:** 10.1186/s13052-024-01620-1

**Published:** 2024-03-07

**Authors:** Roberto Rondelli, Tamara Belotti, Riccardo Masetti, Franco Locatelli, Maura Massimino, Alessandra Biffi, Carlo Dufour, Franca Fagioli, Giuseppe Menna, Andrea Biondi, Claudio Favre, Marco Zecca, Nicola Santoro, Giovanna Russo, Silverio Perrotta, Andrea Pession, Arcangelo Prete

**Affiliations:** 1 Pediatric Oncology and Hematology Unit “Lalla Seràgnoli, IRCCS Azienda Ospedaliero-Universitaria Di Bologna, Bologna, Italy; 2 Department of Hematology Oncology and Transfusion Medicine, IRCCS Pediatric Hospital “Bambino Gesù”, Rome, Italy; 3 Pediatric Unit, Fondazione IRCCS Istituto Nazionale Dei Tumori, Milan, Italy; 4 Pediatric Hematology, Oncology and Stem Cell Transplant Division, Padua University Hospital, Padua, Italy; 5 Department of Pediatric and Hemato-Oncologic Sciences, IRCCS “Istituto Giannina Gaslini”, Genoa, Italy; 6 Paediatric Onco-Haematology Division, Regina Margherita Children’s Hospital, City of Health and Science of Turin, Turin, Italy; 7 Department of Woman, Child and of General and Specialized Surgery, Pediatric Hematology Unit, Università Degli Studi Della Campania, Naples, Italy; 8 Pediatrics, Fondazione IRCCS San Gerardo Dei Tintori, Monza, Italy; 9 School of Medicine and Surgery, University of Milano-Bicocca, Monza, Italy; 10 Department of Pediatric Hematology Oncology, Meyer Children’s Hospital Istituto Di Ricovero E Cura a Carattere Scientifico (IRCCS), Florence, Italy; 11 Pediatric Hematology/Oncology, Fondazione Istituto Di Ricerca E Cura a Carattere Scientifico (IRCCS) Policlinico S. Matteo, Pavia, Italy; 12 Oncology and Hematology, University Hospital of Policlinic, Bari, Italy; 13 Department of Clinical and Experimental Medicine, Pediatric Hematology and Oncology Unit, University of Catania, Catania, Italy; 14 Department of Women, Children and General and Specialized Surgery, “Luigi Vanvitelli” Università Degli Studi Della Campania, Naples, Italy; 15 Pediatric Unit, IRCCS Azienda Ospedaliero-Universitaria Di Bologna, Bologna, Italy

**Keywords:** Paediatric, Haematology, Oncology, Migration

## Abstract

**Background:**

In Italy, there is a network of centres headed by the Italian Association of Pediatric Hematology and Oncology (AIEOP) for the diagnosis and treatment of paediatric cancers on almost the entire national territory.

Nevertheless, migration of patients in a hospital located in a region different from that of residence is a widespread habit, sometimes motivated by several reasons.

The aim of this paper is to assess the impact of migration of children with cancer to AIEOP centres in order to verify their optimal distribution throughout the national territory.

**Methods:**

To this purpose, we used information on 41,205 registered cancer cases in the database of Mod.1.01 Registry from AIEOP centres, with age of less than 20 years old at diagnosis, diagnosed from 1988 to 2017.

Patients’ characteristics were analysed and compared using the X^2^ or Fisher’s exact test or Mann–Whitney test, when appropriate.

Survival distributions were estimated using the method of Kaplan and Meier, and the log-rank test was used to examine differences among subgroups.

**Results:**

Extra-regional migration involved overall 19.5% of cases, ranging from 23.3% (1988–1997) to 16.4% (2008–2017) (*p* < 0.001).

In leukaemias and lymphomas we observed a mean migration of 8.8% overall, lower in the North (1.2%) and Centre (7.8%) compared to the South & Isles (32.3%).

In the case of solid tumours, overall migration was 25.7%, with 4.2% in the North, 17.2% in the Centre and 59.6% in the South & Isles.

For regions with overall levels of migration higher than the national average, most migration cases opted for AIEOP centres of close or even neighbouring regions.

Overall survival at 10 years from diagnosis results 69.9% in migrants vs 78.3% in no migrants (*p* < 0.001).

**Conclusions:**

There is still a certain amount of domestic migration, the causes of which can be easily identified: migration motivated by a search for high specialization, migration due to lack of local facilities, or regions in which no AIEOP centres are present, which makes migration obligatory.

Better coordination between AIEOP centres could help to reduce so-called avoidable migration, but technical and political choices will have to be considered, with the active participation of sector technicians.

## Introduction

The term health migration commonly refers to hospitalization in a hospital located in a region (or country) different from that of residence, and represents a rather significant phenomenon in quantitative terms, in fact it affects 8.8% of the Italian child population, value slightly higher than that relating to the whole population (7.7%) [1].

It is also a phenomenon that affects health planning, being an indicator of the Essential Levels of Assistance (LEAs) that the regions are required to ensure and a possible indicator of inequality in access to services [2].

Healthcare migration can be classified into 3 categories: migration motivated by objective healthcare reasons (highly specialized centres, or for the treatment of rare diseases such as paediatric tumours); basic migration due to geographical and road needs as well as family or historical roots (migration from the southern regions to the central-north), and finally avoidable migration, the causes of which are to be found in a lack (also of information) or inefficiency of local structures with relative distrust of users.

In a 2016 publication by the Italian Center for Social Investment Studies (Censis), regarding the central aspect of motivations and causal factors that substantiate the choice of admission to a hospital other than one’s own region, three main reasons emerge, or rather three different ones questions/expectations from patients. These reasons have obviously origin in areas that overlap, as it is never possible to trace a complex choice like this for only one reason.

A first indication that emerges from the data is that in most cases the choice to migrate not exclusively attributable to the area of “necessity”, and therefore to the lack of adequate structures in the area or to their limited accessibility as one might suppose (sometimes the health migration is associated with the image of the so-called "journey of hope"), but instead mostly constitutes a search for quality.

The second area of motivation that emerged concerns the practical-logistic dimension, i.e. the knowledge of the doctor or a nurse in the hospital (20%), the greater ease of reaching the structure (6%) or the presence of a family member in the area (5%). The third area of motivation has to do with the dimension of “necessity" that is, pertains to the impossibility (or to the difficulty due to the excessive waiting time) to carry out the type of services required [3].

Finally, it should be remembered that medical migration does not always and does not necessarily represent a negative or worrying phenomenon.

In some cases, such as the diagnosis and treatment of rare diseases such as pediatric cancers, or in the case of services that require an adequate volume of cases to ensure quality and efficiency, it is reasonable to favor the concentration of patients in specialized reference structures.

This is what happens in Italy, in the case of the diagnosis and treatment of pediatric cancers, where there is a network of centres headed by the Italian Association of Pediatric Hematology and Oncology (AIEOP).

Currently these centres are 54, present on almost the entire national territory (except in Valle d'Aosta, Molise and Basilicata) half of which are located in Northern Italy (26), 13 in the Centre and 15 in the South & Isles area.

One of the objectives of the Mod.1.01 form, used since 1 January 1989 by the AIEOP centres for the registration of all newly diagnosed cases of malignant tumors in childhood, was to evaluate extra-regional migration for diagnosis and treatment in an AIEOP centre in a region other than that of residence, as an indicator reflecting the situation of assistance/offer from the network of AIEOP centres [4–6].

A secondary objective was to create a common capture form for all cases recruited by an AIEOP centre entered or not an official AIEOP protocol, according to the online electronical system adopted by AIEOP and shared by all adherent centres [7].

The online database recorded not only anagraphical data, but basic clinical and therapeutic ones, together with facilities for adjournment of follow-up status.

We therefore, wanted to use the database of Mod.1.01 Registry to assess the impact of migration of children with cancer to AIEOP centres of regions other than that of residence in order to verify the capillarity of distribution of the AIEOP centres throughout the national territory in an adequate way and to quantify the residual migration amount historically linked to some areas of our Country.

## Patient and methods

### Patients and sample

To this purpose, we used information on all registered cancer cases in the database of Mod.1.01 Registry from AIEOP centres, with age of less than 20 years old at diagnosis, diagnosed from 1.1.1988 to 31.7.2017.

### Statistical analysys

Data were analysed as of July 31, 2017. All data were stored in a central database (Mod.1.01 Registry), and were processed at the AIEOP Operation Office [8].

Patients’ characteristics, such as recruitment by year, type of disease, age at diagnosis, geographical area of origin, distribution on the Italian territory, and treatment centre, were analysed and compared, when appropriate, using the X2 or Fisher’s exact test in the case of discrete variables, or the Mann–Whitney test in the case of continuous variables.

95% Confidence Interval (CI) of mean or percentage were reported in case of statistically significant difference.

Overall survival (OS) was computed from the date of diagnosis to the date of death from any cause, or the last date of contact, if still alive.

Survival distributions were estimated using the method of Kaplan and Meier, and the log-rank test was used to examine differences among subgroups [9].

Results were expressed as cumulative probability (%) and standard error (SE).

All *p* values are 2-sided and values less than 0.05 were considered statistically significant.

Statistical analysis was carried out using the Stata statistical package, version 7.0 [10].

## Results

### Patient migration in AIEOP centres

The analysis of the migratory phenomenon concerned all cases aged 0–19 years enrolled by AIEOP centres. Analysing the 41,205 cases affected by malignant tumours diagnosed by the AIEOP centres in the period 1.1.1988–31.7.2017, extra-regional migration involved overall 19.5% (8023/41,205) of cases: 23.3% (2513/10777) in the ten-year period 1988–1997, 20.1% (2808/13964) during 1998–2007 and 16.4% of cases (2702/16464) in the following 9 years and 7 months period (January 2008—July 2017) thus recording a significant reduction (*p* < 0.001) in all Italian zone from the first to the last period considered.

Regarding class of age of diagnosis, extra-regional migration involved 19.2% (7081/36885) of cases aged < 14 years and 21.0% (1328/6332) of cases with 15–19 years of age (*p* = 0.007).

Extra-regional migration has been reduced over the years in all classes of age at diagnosis. In the case of cases of 0–14 years at diagnosis, resulted of 23.2% (2373/10249) in the period 1988–1997, 20.0% (2538/12615) in 1998–2007 and 15.8% (2220/14021) in 2008-July 2017 (*p* = 0.000).

Likewise, extra-regional migration has been reduced over the years even in cases aged 15–19 years at diagnosis: 26.6% (259/974) in the period 1988–1997, 22.2% (445/2003) in 1998–2007 and 18.6% (624/3355) in 2008-July 2017 (*p* = 0.000).

Overall, 9 Italian regions presented a higher value than the national average, headed by Trentino Alto Adige with about 90% of resident cases migrated in centres of another region, while the lowest value was observed in Lombardy (7.5%) and Liguria (9.7%) among the northern regions, and Lazio (7.7%) and Tuscany (16.0%) among the central regions.

**Fig. 1:**
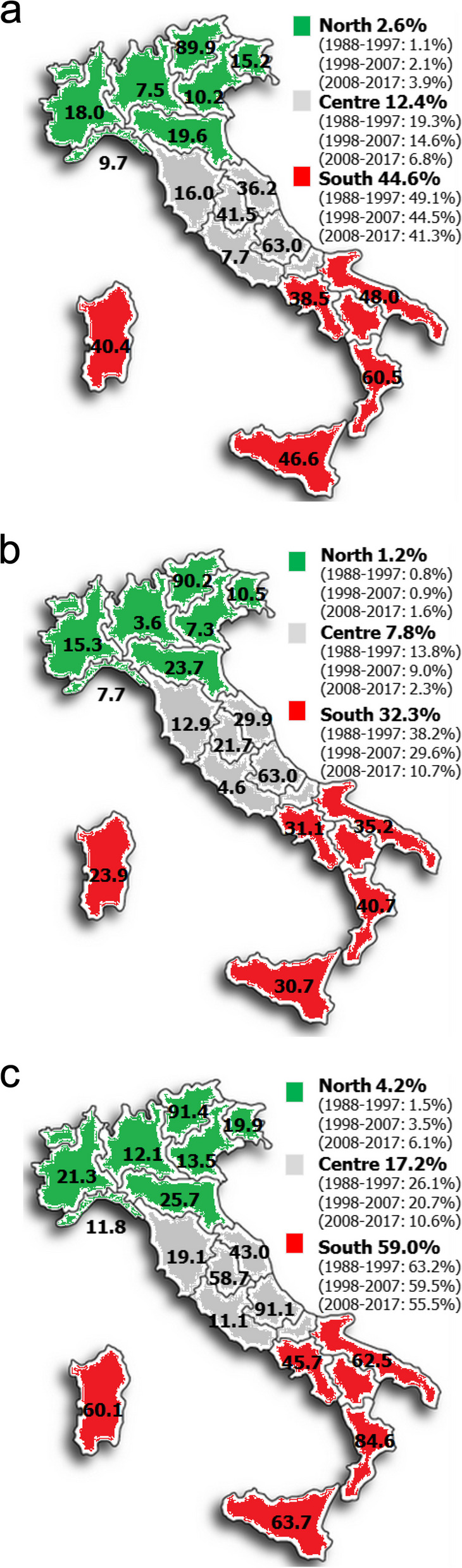
Extra-regional migration by region of residence: overall (**a**), for Leukaemia-lymphomas (**b**), for Solid tumours (**c**)

While in southern and island regions migration ranged from 37.5% in Campania to 60.5% in Calabria, Abruzzo is the Italian region with the maximum value equal to 63.0% (Fig. 1a).

Considering single types of disease, in the case of leukaemias and lymphomas (LL) we observed a mean migration in the period 1988–2017 of 8.8% (2760/19899) overall, lower in the North (99/8487: 1.2%) and Centre (323/4166: 7.8%) compared to the South & Isles (2338/7246: 32.3%) (Fig. 1b).

In cases of leukaemias and lymphomas of age < 15 years at diagnosis the mean migration observed was 19.2% (7081/36885) overall, lower in the North (422/15801: 2.7%) and Centre (957/7929: 12.1%) compared to the South & Isles (5702/13155: 43.4%) (*p* = 0.000), while in cases 15–19 years of age was 21.0% (1328/6332) overall, lower in the North (50/2861: 1.7%) and Centre (213/1505: 14.1%) compared to the South & Isles (1065/1966: 54.2%) (*p* = 0.000).

In the case of solid tumours (ST), overall average migration was 25.7% (4892/19005), with 4.2% (344/8273) in the North, 17.2% (736/4275) in the Centre, while in the South & Isles it involved almost 60% (3849/6457: 59.6%) of cases, which is, in any case, a lower percentage than in the previous years period (Fig. 1c).

Regarding solid tumours of age < 15 years at diagnosis the mean migration observed was 25.4% (4303/16946) overall, lower in the North (321/7277: 4.4%) and Centre (609/3807: 16.0%) compared to the South & Isles (3373/5862: 57.5%) (*p* = 0.000), while in cases 15–19 years of age was 28.6% (589/2059) overall, lower in the North (23/996: 2.3%) and Centre (127/468: 27.1%) compared to the South & Isles (439/595: 73.8%) (*p* = 0.000).

It is worth noting that if we consider the regional level, migration is equal to 19.5% (8023/41205), but if instead we consider the geographical area of residence (as a geographical limit), we see that this drops to 7.0% (2879/41205) (North: 2068/17753 = 11.6%, Centre: 635/9007 = 7.1%, South & Isles: 176/14445 = 1.2%).

Analysing the data for each geographical area, the extra-area migration is 2.6% (459/17753) in the North and 12.4% (1116/9007) in the Centre, and therefore more than 3/4 (80%) of the migration to the North and less than 1/2 (36%) to the Centre takes place in centres of the same geographical area. If we analyse migration flows for each of the 9 regions with overall levels of migration higher than the national average, we observe that most migration cases resident in Trentino Alto Adige (74%) migrated to AIEOP centres in nearby Veneto, as likewise patients from Umbria (20%) and Abruzzo (30%) sought out AIEOP centres in neighbouring Lazio. Even patients resident in Campania (13%), Puglia (13%), and Calabria (22%), mostly migrated to AIEOP centres in Lazio (between 13 and 22%), which is a close or even neighbouring region.

In the three regions where no AIEOP centres are present, migration occurred mainly towards AIEOP centres in neighbouring or nearby regions. In Valle d’Aosta 82% (73/89) of cases migrated to Piedmont and 11% (10/89) to Liguria, from Molise 49% (103/212) of cases moved to Lazio and 26% (56/2012) to Abruzzo, from Basilicata 33% (139/416) of cases moved to Puglia and 31%(130/416) to Lazio.

On the other hand, there are also distant migration flows. Between 7 and 13% of patients resident in Campania (7.8%), Puglia (12%), and Calabria (13%) sought care in AIEOP centres in Lombardy; in Sicily (14%) and Sardinia (16%) most residents who sought care elsewhere (14–16%) went to the AIEOP centre in Liguria.

**Fig. 2:**
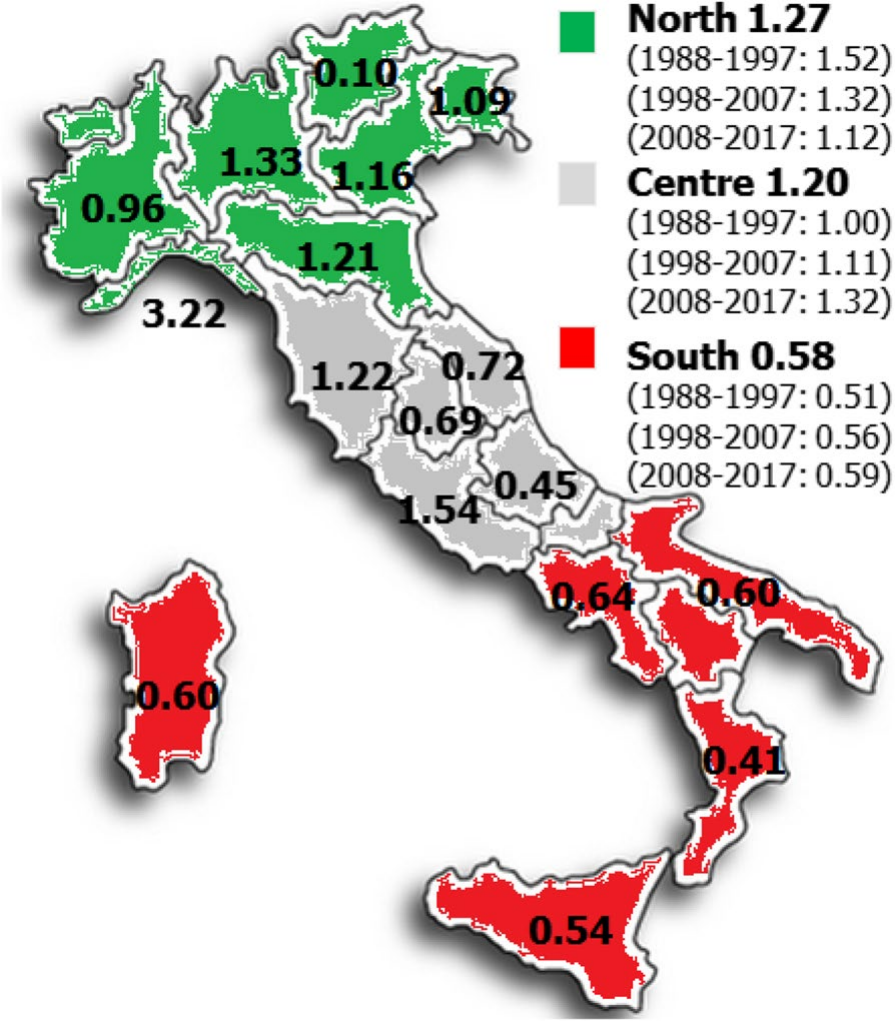
Treated cases – resident cases ratio by region of treatment

Overall, the ability to cope with a burden of treatment involving more patients than those in their catchment area, also expressed as the ratio of cases treated to resident cases, was higher in some northern regions, especially Liguria, Lombardy, Veneto, and central regions (Tuscany and Lazio): these regions thus proved to have centres with greater accessibility (Fig. 2).

### Patient migration and survival

The results relating to migrated patients, in order of survival, are globally lower in these than in those who have turned to centres in their geographical area.

**Fig. 3:**
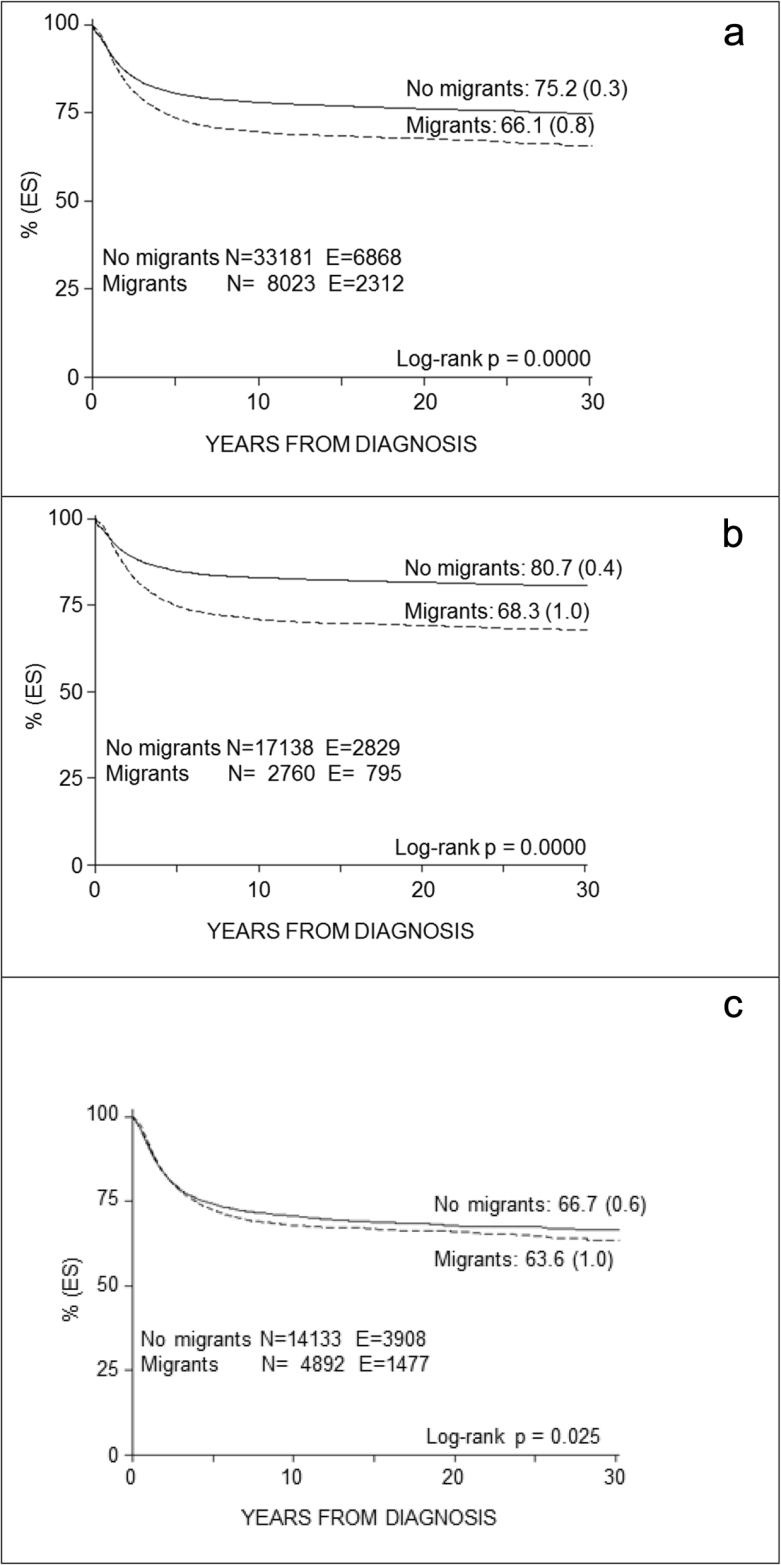
Overall survival (**a**), for Leukaemia-lymphomas (**b**), for Solid tumours (**c**)

In fact, overall survival (SE) at 10 years from diagnosis results 69.9% (0.5) in migrants vs 78.3% (0.2) in no migrants (*p* < 0.001) (Fig. 3a).

A significant difference results between migrants vs no migrants in each 10-years period and considering disease: 10-years survival (SE) for leukaemias-lymphomas: 70.9% (0.9) for migrants vs 82.9% (0.3) for no migrants (*p* < 0.001); for solid tumours: 68.0% (0.7) for migrants vs 70.6% (0.4) for no migrants (*p* = 0.025) (Fig. 3b,c).

Considering each type of diagnosis, 10-years survival (SE) for migrants vs no migrants resulted in 65.2% (1.1) vs 80.2% (0.4) for leukaemias (*p* < 0.001); 83.2% (1.3) vs 89.4% (0.4) for lymphomas (*p* < 0.001); 65.5% (1.2) vs 63.3% (0.8) for CNS tumours (*p* = 0.028); 60.8% (2.0) vs 67.8% (1.0) for neuroblastoma (p < 0.01); 94.5% (1.3) vs 93.1% (1.4) for retinoblastoma (*p* = 0.19); 83.2% (2.1) vs 86.9% (0.9) for renal tumours (*p* = 0.13); 75.1% (4.7) vs 66.2% (2.8) for hepatic tumours (*p* = 0.06).

70.9% (0.9) for migrants vs 82.9% (0.3) for no migrants (*p* < 0.001); for solid tumours: 68.0% (0.7).

It was 55.2% (2.0) vs 59.6% (1.4) for malignant bone tumours (*p* = 0.19); 63.0% (2.1) vs 67.8% (1.1) for soft tissue sarcomas (*p* = 0.07); 84.0% (2.4) vs 89.5% (1.0) for germ cell tumours, and neoplasms of gonad (*p* = 0.024); 82.6% (3.7) vs 87.8% (1.6) for other malignant epithelial neoplasms and malignant melanomas (*p* = 0.21); 74.3% (10.0) vs 76.5% (5.0) in other and unspecified malignant neoplasms (*p* = 0.44).

## Discussion

AIEOP represents the Italian network for pediatric haematology-oncology diagnosis and treatment, demonstrated by the overall accrual of cases during a thirty years period analysed: about 1400/year (0–14 years: 1377/1400 year = 98.3% expected) and about 250/year (15–19 years: 232/770 year = 30.0% expected) [11], the latter very close to what already previously reported by AIEOP [12].

This difference by age group depend on the fact that AIEOP centres were born at the beginning for diagnosis and treatment of children (age 0–14 years) and only subsequently, enrollment was extended to adolescents and young adults (15–19 years) due to administrative rules involved Italian pediatric centres from the end of the 90 s.

This fact has contribute to arise the observed/expected ratio for adolescents from 10% in 1989–2006 to 28% in 2007–2012 and finally to 37% in 2013–2017 [12].

Migration to centres in other geographical areas may be the result of a local lack of structures or even of the belief that better results can be obtained then those assumed, often erroneously, in the nearest centres.

Many could be the reasons of this phenomenon, sometimes historical, such as the south vs north migration, some other due to a major visibility of some centres in media channels. Sometimes these opportunities are real, such as accessibility to specific services (surgery, proton therapy, transplants), some other much is due to media involvement that can push towards some centres and not others.

However, extra-regional migration for diagnosis and/or treatment of pediatric cases declined significantly over time (from 29.3% (3159/10777) 1988–1997 to 26.8% (3741/13964) 1998–2007 to 22.2% (3651/16464) 2008–2017; *p* < 0.001), perhaps due to an improvement in the organization of centres, cooperation with family paediatricians, and greater trust of families in local centres.

This happened both in children and young adults. In fact, we have found a significant reduction over the years for each group, even if this phenomenon is still present in our country.

In the last period analysed (2008–2017) the migratory phenomenon involved an average of 16.4% (2702/16464) of patients residing in the 17 italian regions where there are AIEOP centres, and is higher in the South & Isles (32%), with peaks exceeding 50% in Trentino Alto Adige (177/212 = 83.5%), Calabria (340/565 = 60.2%) and Abruzzo (197/339 = 58.1%).

This phenomenon results in a recent analysis performed on passive mobility and related costs between geographical areas of Italy regarding 2019. In this study resulted that out-region hospitalization ranged from 6.9% for children living in the Centre-North vs 11.9% for children living in the South & Isles.

The difference is even greater in case of high complexity hospitalizations, such as malignancies, confirmed by an extra-regional migration significantly lower for leukaemias and lymphomas than in solid tumours (*p* = 0.000).

In the latter case, children living in the South & Isles were more frequently treated in other regions than those living in the Centre-North (21.3% vs 10.5%) [1], even if lower than that recorded in previous years by the AIEOP.

Considering results of migrants in terms of survival, there is a statistically significant lower value both globally and for most pathologies.

These results could be due in part to migration in advanced stage of disease and difficulty in managing inpatients and their families far from home.

Therefore, migration seems to be justified by better survival of migrants affected by leukaemias, lymphomas, CNS tumours, germ cell tumours, trophoblastic tumours, and other neoplasms of gonads, while there are no statistical differences in the other types of neoplasms.

However survival calculated by AIEOP is very close to what published by AIRTUM (5 years survival: 79% vs 78% for 0–14 years old and 76% vs 82% for 15–19 years old) [13] and also consent to verify the best outcome of patients treated by multicenter AIEOP protocols vs other therapies (5 years survival: 81% vs 74%, *p* = 0.000).

Another topic that should be analysed is represented by patients that seek services from hospitals outside the AIEOP network. It could be important to know the number of cases in which a non-protocol or non-paediatric oncology guided treatment could have impaired the outcome.

Migration abroad for the treatment of childhood cancer is however a no longer frequently observed phenomenon in our Country [13].

In fact, about only 3 cases/year results born in Italy, but emigrated abroad in 1999–2007 [14], while 25% of adolescents are supposed to address the adult centers [15].

There is still a certain amount of domestic migration, the causes of which can be easily identified. Migration is motivated by the search for higher specialization, which drives cases towards centres specialized in particular diseases. Migration can be due to the lack of local facilities, either in part, as in Trentino Alto Adige, Calabria and Abruzzo, or completely, as in Valle d’Aosta, Molise and Basilicata, the three regions in which no AIEOP centre is present.

There is still a significant and residual component of "elective" migration, which mainly affects patients residing in the South & Isles, motivated by organizational shortcomings that have created a "historical rooted distrust" toward health centres out of their home regions [16].

## Conclusions

On these phenomena, only partly motivated by residual objective deficiencies, the AIEOP does not currently have the tools to effectively intervene.

Better coordination between AIEOP centres could help to reduce so-called avoidable migration.

A solution to the problem likely lies in the planning of interventions aimed at defining a more homogeneous national paediatric oncological network, which guarantees patients the highest possible quality, within at least a "macro-regional" area which reduces motivated migratory flows.

The current corporate public health organization could create however interests in conflict with this "virtuous" path, to the extent that oncology and haematological structures may be considered "excellent" and "productive" because of their ability to attract patients from other regions.

In this complex and delicate context, technical and political choices will have to be considered and interpenetrated, hopefully with the active participation of sector technicians.

## Data Availability

The datasets used and/or analysed during the current study are available from the corresponding author on reasonable request.
